# Supplementary material for: Arabidopsis α-Aurora kinase plays a role in cytokinesis through regulating MAP65-3 association with microtubules at phragmoplast midzone
Source: Nat Commun. 2024 May 6;15:3779. doi: 10.1038/s41467-024-48238-9 (PMC11074315; doi:10.1038/s41467-024-48238-9)
Supplement: Supplementary file 1 — Supplementary Information [file 41467_2024_48238_MOESM1_ESM.pdf]

Supplementary Information

Arabidopsis α-Aurora Kinase Plays a Role in Cytokinesis through Regulating MAP65-3 Association with Microtubules at Phragmoplast Midzone

Xingguang Deng [1, a, b], Yu Xiao[1, b], Xiaoya Tang [1], Bo Liu[2, a], Honghui Lin [1, a]

[1] Key Laboratory of Bio-resource and Eco-environment of Ministry of Education, College of Life Sciences, State Key Laboratory of Hydraulics and Mountain River Engineering, Sichuan University, Chengdu, 610064, China

[2] Department of Plant Biology, College of Biological Sciences, University of California, Davis, CA 95616, USA

[a] Address correspondence to: xgdeng@scu.edu.cn, bliu@ucdavis.edu, hhlin@scu.edu.cn

[b] Authors contributed equally to this work

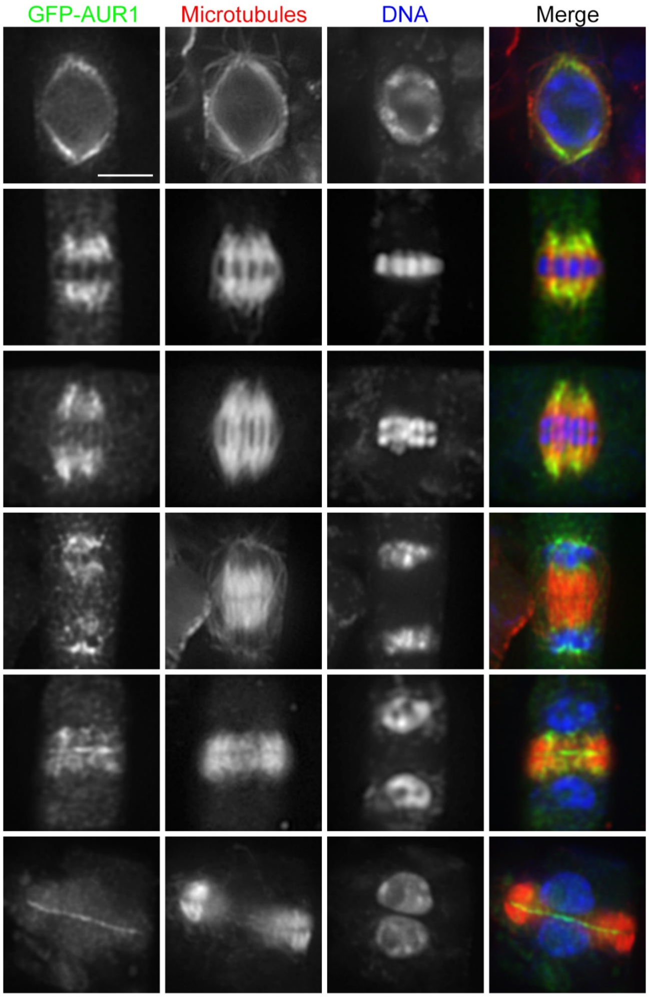

Supplementary Fig. S1: Localization of GFP-AUR1 throughout the cell cycle in Arabidopsis. The merged images have GFP-AUR1 detected by the anti-GFP antibody in green, microtubules in red and DNA in blue. Micrographs are representative of more than 100 cells from three independent lines with similar results. Bars, 5 µm.

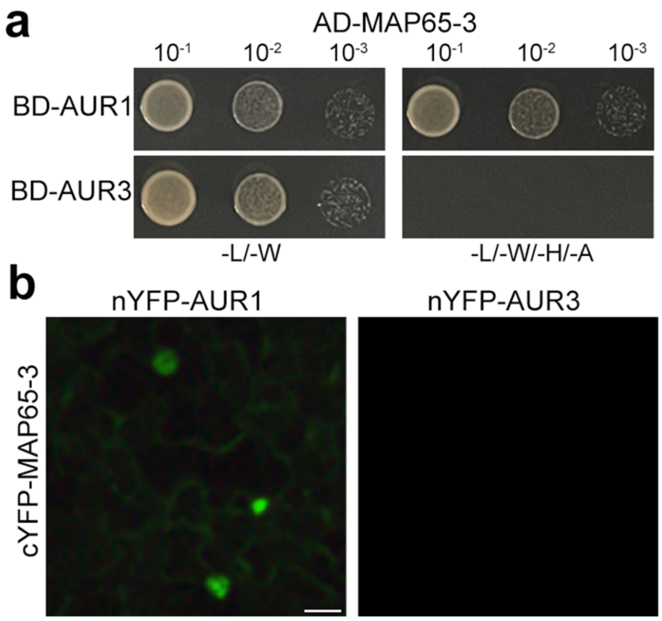

Supplementary Fig. S2: Arabidopsis MAP65-3 interacts with α-Aurora but not β-Aurora. a Y2H assay to examine interactions between MAP65-3 and different Aurora members. The yeast cultures were spotted on interaction-selective (-L/-W/-H/-A, right column) and vector-selective (-L/-W, left column) media and photographed after incubation at 30°C for 2 days. **b** BIFC assay of the interactions of MAP65-3 (fused with the C-terminal fragment of YFP) with AUR1 and AUR3 (fused with the N-terminal fragment of YFP) in *N. benthamiana*. The experiments were repeated three times with similar results. Bars, 10 μm.

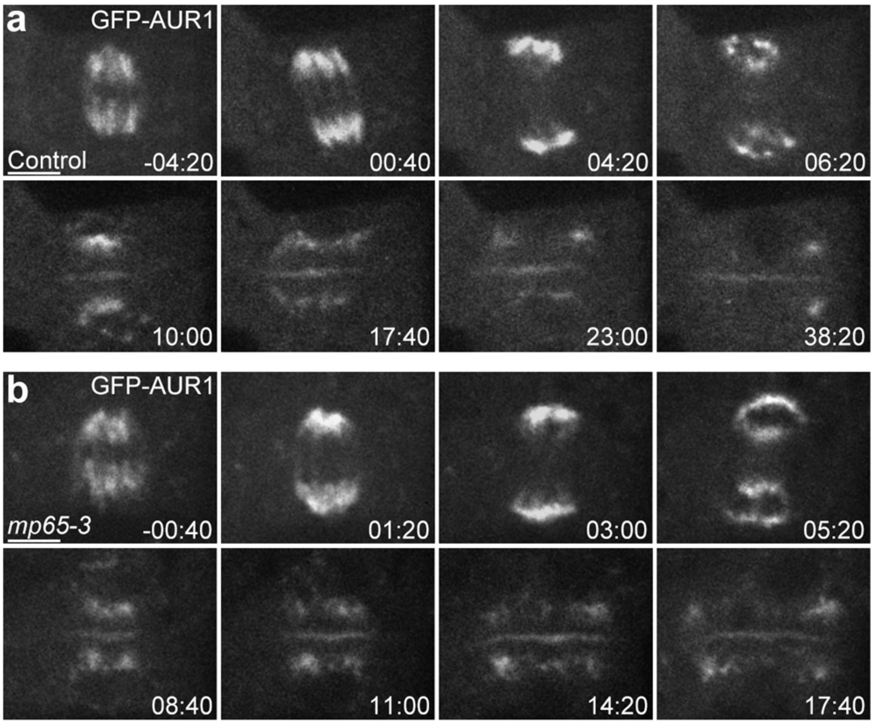

Supplementary Fig. S3: Live-cell imaging of GFP-AUR1 in *aur1 aur2* (control, a) and *map65-3* (b) plants. Images are acquired from Supplementary Movie S3-S4. Similar results were obtained in three independent transformed lines. Bars, 5 µm.

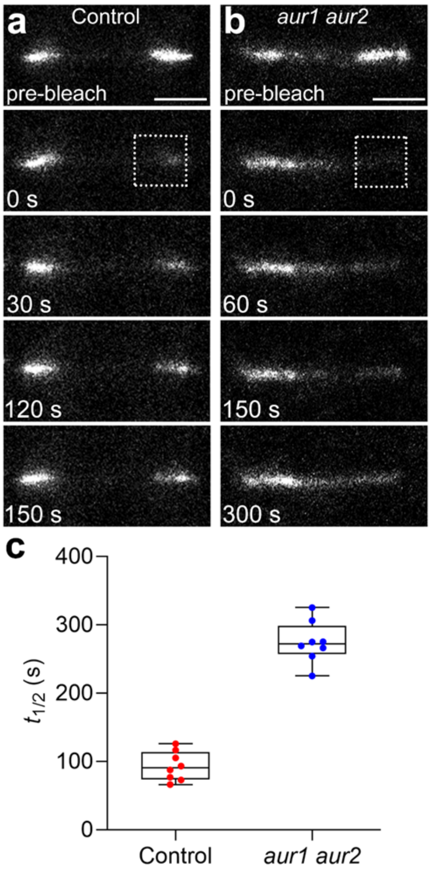

Supplementary Fig. S4: Delayed MAP65-3 turnovers at late cytokinesis in *aur1 aur2* plants. a-b FRAP analysis of MAP65-3-GFP in *map65-3* (control, **a**) and *aur1 aur2* (**b**) backgrounds. Bleach regions are indicated by a dashed square in first postbleach images. Bars, 5 µm. **c** Quantification of the half-life ($t_{1/2}$) of the fluorescence recovery after bleaching in control and *aur1 aur2* cells (n=8 cells). Data are presented as box-and-whisker plots with individual points, showing the interquartile range (box), the median (horizontal line), and minimum and maximum values (whiskers).

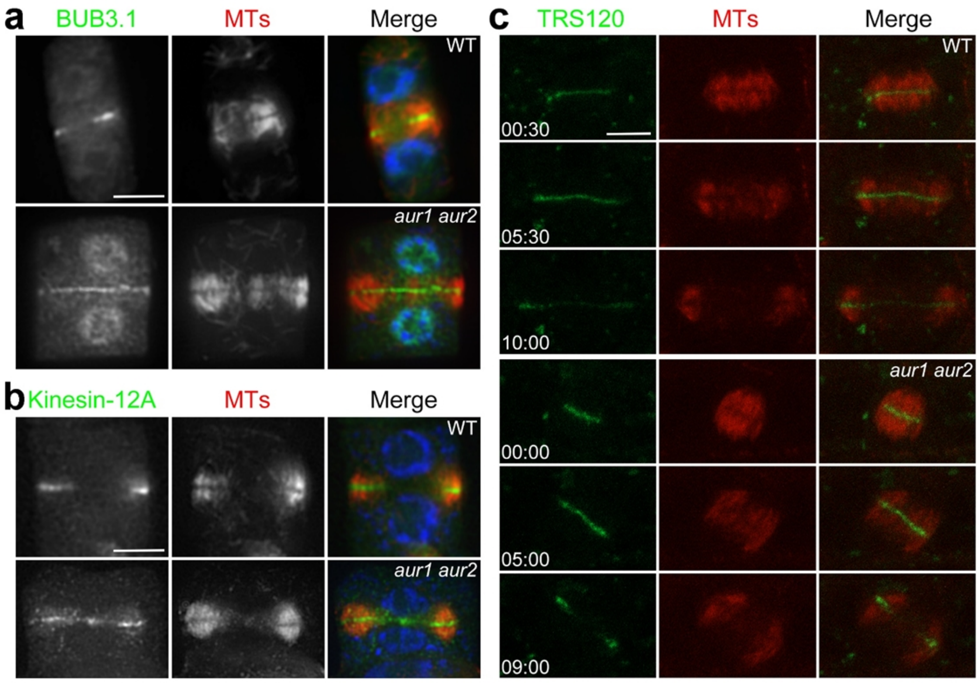

Supplementary Fig. S5: Localization of BUB3.1 (a), Kinesin-12A (b) and TRS120 (c) in WT and *aur1 aur2* plants. Cells in panel **a**-**b** were detected through immunofluorescence staining, cells in panel **c** were examined by live-cell imaging. Similar results were obtained in three independent transformed lines. Bars, 5 µm.

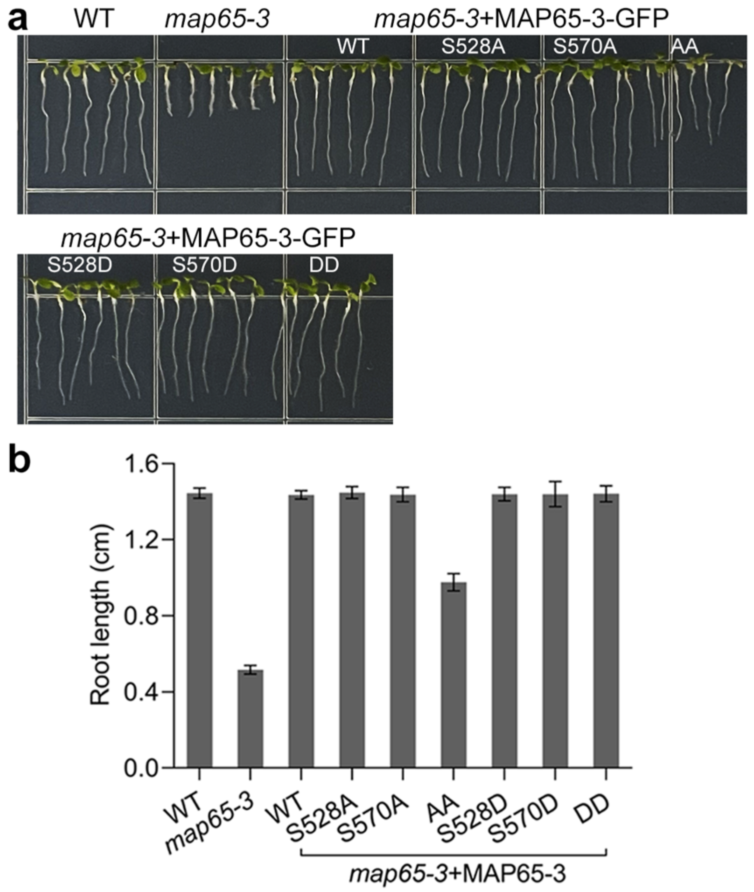

Supplementary Fig. S6: Expression of the non-phosphorylatable MAP65-3 partially restores the growth defects caused by the *map65-3* mutation. a Seedling growth patterns of the wild-type, *map65-3* mutant, and *map65-3* mutant expressing wild-type, various phospho-defective and phospho-mimicking forms of MAP65-3. **b** Quantification of root lengths in the seedlings in panel **a**, data are means ± SD measured from 6 individual seedlings (n=6 plants).

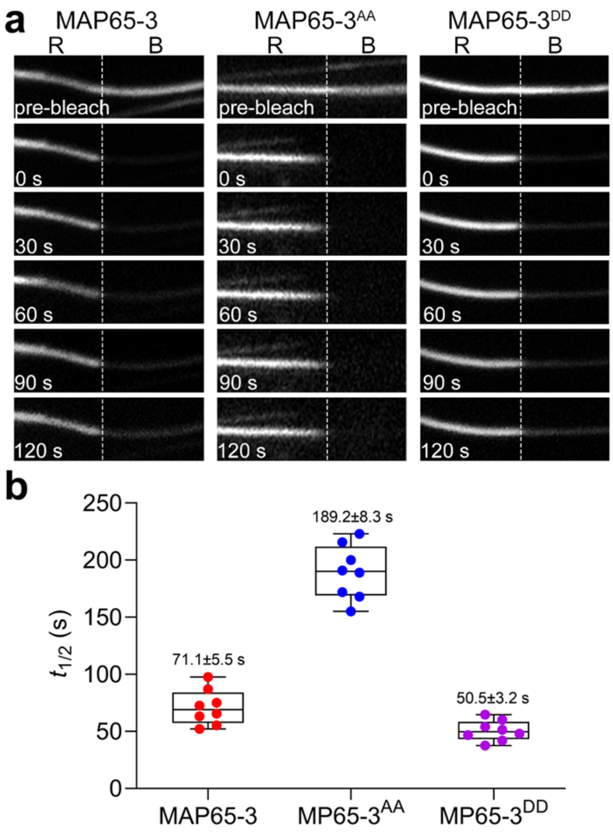

Supplementary Fig. S7: Aurora-mediated phosphorylation promotes MAP65-3 dissociating from microtubules. a FRAP analysis of MAP65-3-GFP, MAP65-3^{AA}-GFP and MAP65-3^{DD}-GFP in *N. benthamiana* leaves. Both signals of the neighboring reference (R) and bleaching-recovery (B) were taken at various time points. **b** Quantification of the fluorescence recovery half-life ($t_{1/2}$) of MAP65-3-GFP, MAP65-3^{AA}-GFP and MAP65-3^{DD}-GFP (n=8 cells). Data are presented as box-and-whisker plots with individual points, showing the interquartile range (box), the median (horizontal line), and minimum and maximum values (whiskers).

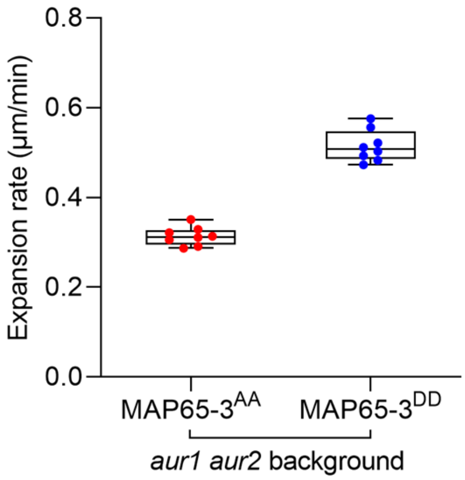

Supplementary Fig. S8: Quantitative assessment of cell plate expansion velocity in *aur1 aur2* plants expressing MAP65-3^{AA} and MAP65-3^{DD} constructs. Data are presented as box-and-whisker plots with individual points (n=8 cells), showing the interquartile range (box), the median (horizontal line), and minimum and maximum values (whiskers).

Supplementary Table S1: Primers used in this study

Primer	Sequence	Information
T-2-7	CAAAAATAGTTTGAGCTGGTTGG	SALK_022166 LP
T-2-8	TCAACTTTCCTCTTTTGCATCTC	SALK_022166 RP
LBb1.3	ATTTTGCCGATTTCGGAAC	BP for SALK lines
gMAP65-3-F	GacaagtttGTACAAAAAAGCAGGCTTTGGAGGTTAAGTTAGCCGAAATAG	Primers for genomic MAP65-3 cloned into pDONR221 via BP reaction
gMAP65-3-R	GaccactttgtacaagAAAGCTGGGTAAACCAAACGACATTCAGACTGTAG	
G-3-15	tggacagcaaatgggtcgcggatccATGGCAAGTGTTCAAAAAGATCC	Primers for MAP65-3 CDS (1-588) gibson into pET28a vector digest with BamHI & SalI
G-3-16	cgagtgcggccgcaagcttgtcgacGGGGGAAAACGGTTTGCGAACTA	
G-3-17	AAGACTTgcaTTAGGAGCAGCAATGCATCAGAC	MAP65-3 S528A mutation primers for reverse PCR
G-3-18	GCTCCTAAtgcAAGTCTTCTGTTAGATGCACCAC	
G-3-19	GGAAACAAgCAATGAATCCTTCTGAAATGTTGC	MAP65-3 S570A mutation primers for reverse PCR
G-3-20	ATTCATTGcTTGTTTCCTTGAGGGTAGTCCTG	
G-4-9	tgtacaaaaaagcaggctccgcgATGGCAAGTGTTCAAAAAGATCCG	Primers for gibson MAP65-3 CDS into pENTR vector by gibson assemble
G-4-10	ttgtacaagaaagctgggtcggcAACCAAACGACATTCAGACTGTAG	
G-4-45	GGGGGAAAACGGTTTGCGAA	R primer with attL2-F to get pEN-MAP65-3 CDS (1-588)
G-4-46	TTGTTCTGCTATGAGCTGTC	R primer with attL2-F to get pEN-MAP65-3 CDS (1-495)
G-4-47	GTCTGACACTGGCAACAAGT	R primer with attL2-F to get pEN-MAP65-3 CDS (1-340)
G-4-48	TTCGGAATGAACAAGTTCTC	R primer with attL2-F to get pEN-MAP65-3 CDS (1-150)
G-5-14	aagcttgtcgacTCAGTCTGACACTGGCAACAAGTGG	R primer to get pET28a-MAP65-3 (1-340) with G-5-9
G-5-15	aagcttgtcgacTCATTCGGAATGAACAAGTTCTCCT	R primer to get pET28a-MAP65-3 (1-150) with G-5-9
G-5-16	aagcttgtcgacTCATTGTTCTGCTATGAGCTGTCCT	R primer to get pET28a-MAP65-3 (1-495) with G-5-9
attL2-F	gCCGACCCAGCTTTCTTGTACAAA	
attL1-R	GCGGAGCCTGCTTTTTTGTACAA	
GST-AUR1-F	atccccgggaatttccggtggtggtggtggaATGGCGATCCCTACGGAGACACA	Primers for AUR1 CDS cloned into the pGEX-KG vector digest with BamHI & EcoRI
GST-AUR1-R	acgatgaataagcttgagctcgagtcgaccTTAAACTCTGTAGATTCCAGAAGGATCA	
G-7-39	TGGGAACCAATTCAGTCGACTGgacCCTCTTCAGTGCCTCAAATGTC	Primers for gTRS120 gibson into pENTR4 digest with BamHI & EcoRI
G-7-40	AGATATCTCGAGTGCGGCCGCGgCAGTGCACCTCCAGCTACACAGAC	
G-6-35	TGGGAACCAATTCAGTCGACTGgtgtgcaagtagttgtgaactttag	Primers for gKinesin12A gibson into pENTR4 digest with BamHI & EcoRI
G-6-36	AGATATCTCGAGTGCGGCCGCGgTATGTTGCACCTATCATAACCCGA	

Description of Additional Supplementary Files

Supplementary Movie S1. Live imaging of cytokinesis in wild-type cells expressing GFP-TUB6.

Supplementary Movie S2. Live imaging of cytokinesis in *aur1 aur2* cells expressing GFP-TUB6.

Supplementary Movie S3. Live imaging of GFP-AUR1 localization during cell division in the *aur1 aur2* background.

Supplementary Movie S4. Live imaging of GFP-AUR1 localization during cell division in the *map65-3* background.

Supplementary Movie S5. FRAP analysis of MAP65-3-GFP in the *map65-3* background.

Supplementary Movie S6. FRAP analysis of MAP65-3-GFP in the *aur1 aur2* background.

Supplementary Movie S7. Rotational views of 3D reconstructed MAP65-3-GFP in *map65-3* cells.

Supplementary Movie S8. Rotational views of 3D reconstructed MAP65-3-GFP in *aur1 aur2* cells.

Supplementary Movie S9. Live-cell imaging of *map65-3* plants expressing MAP65-3-GFP and mCherry-TUB6.

Supplementary Movie S10. Live-cell imaging of *aur1 aur2* plants expressing MAP65-3-GFP and mCherry-TUB6.

Supplementary Movie S11. Live-cell imaging of MAP65-3-GFP in roots of *aur1 aur2* seedlings co-stained with FM4-64.

Supplementary Movie S12. FRAP analysis of MAP65-3^{AA}-GFP in the *map65-3* background.

Supplementary Movie S13. FRAP analysis of MAP65-3^{DD}-GFP in the *map65-3* background.

Supplementary Movie S14. Live-cell imaging of *map65-3* plants expressing MAP65-3^{AA}-GFP and mCherry-TUB6.

Supplementary Movie S15. Live-cell imaging of *map65-3* plants expressing MAP65-3^{DD}-GFP and mCherry-TUB6.

Supplementary Movie S16. Live-cell imaging of MAP65-3^{AA}-GFP in roots of *aur1 aur2* seedlings co-stained with FM4-64.

Supplementary Movie S17. Live-cell imaging of MAP65-3^{DD}-GFP in roots of *aur1 aur2* seedlings co-stained with FM4-64.

Supplementary Data S1. Mass spectrometry of MAP65-3 peptides for *in vitro* kinase assays.
