# Supplementary material for: Arabidopsis α-Aurora kinase plays a role in cytokinesis through regulating MAP65-3 association with microtubules at phragmoplast midzone
Source: Nat Commun. 2024 May 6;15:3779. doi: 10.1038/s41467-024-48238-9 (PMC11074315; doi:10.1038/s41467-024-48238-9)
Supplement: Supplementary file 3 — Description of Additional Supplementary Files [file 41467_2024_48238_MOESM3_ESM.pdf]

Description of Additional Supplementary Files:

Supplementary Data 1: Mass spectrometry of MAP65-3 peptides for in vitro kinase assays

Supplementary Movie 1: Live imaging of cytokinesis in wild-type cells expressing GFP-TUB6.

Supplementary Movie 2: Live imaging of cytokinesis in aur1 aur2 cells expressing GFP-TUB6.

Supplementary Movie 3: Live imaging of GFP-AUR1 localization during cell division in the aur1 aur2 background.

Supplementary Movie 4: Live imaging of GFP-AUR1 localization during cell division in the map65-3 background.

Supplementary Movie 5: FRAP analysis of MAP65-3-GFP in the map65-3 background.

Supplementary Movie 6: FRAP analysis of MAP65-3-GFP in the aur1 aur2 background.

Supplementary Movie 7: Rotational views of 3D reconstructed MAP65-3-GFP in map65-3 cells.

Supplementary Movie 8: Rotational views of 3D reconstructed MAP65-3-GFP in aur1 aur2 cells.

Supplementary Movie 9: Live-cell imaging of map65-3 plants expressing MAP65-3-GFP and mCherryTUB6.

Supplementary Movie 10: Live-cell imaging of aur1 aur2 plants expressing MAP65-3-GFP and mCherryTUB6.

Supplementary Movie 11: Live-cell imaging of MAP65-3-GFP in roots of aur1 aur2 seedlings co-stained with FM4-64.

Supplementary Movie 12: FRAP analysis of MAP65-3AA-GFP in the map65-3 background.

Supplementary Movie 13: FRAP analysis of MAP65-3DD-GFP in the map65-3 background.

Supplementary Movie 14: Live-cell imaging of map65-3 plants expressing MAP65-3AA-GFP and mCherryTUB6.

Supplementary Movie 15: Live-cell imaging of map65-3 plants expressing MAP65-3DD-GFP and mCherryTUB6.

Supplementary Movie 16: Live-cell imaging of MAP65-3AA-GFP in roots of aur1 aur2 seedlings co-stained with FM4-64.

Supplementary Movie 17: Live-cell imaging of MAP65-3DD-GFP in roots of aur1 aur2 seedlings co-stained with FM4-64.
